# Evaluation of multi-assay algorithms for cross-sectional HIV incidence estimation in settings with universal antiretroviral treatment

**DOI:** 10.1186/s12879-022-07850-0

**Published:** 2022-11-11

**Authors:** Wendy Grant-McAuley, Oliver Laeyendecker, Daniel Monaco, Athena Chen, Sarah E. Hudelson, Ethan Klock, Ron Brookmeyer, Douglas Morrison, Estelle Piwowar-Manning, Charles S. Morrison, Richard Hayes, Helen Ayles, Peter Bock, Barry Kosloff, Kwame Shanaube, Nomtha Mandla, Anneen van Deventer, Ingo Ruczinski, Kai Kammers, H. Benjamin Larman, Susan H. Eshleman

**Affiliations:** 1 Department of Pathology, Johns Hopkins University School of Medicine, Baltimore, MD USA; 2 Laboratory of Immunoregulation, National Institute of Allergy and Infectious Diseases, National Institutes of Health, Baltimore, MD USA; 3 Department of Medicine, Johns Hopkins University School of Medicine, Baltimore, MD USA; 4 Institute for Cell Engineering, Johns Hopkins University School of Medicine, Baltimore, MD USA; 5 Department of Biostatistics, Johns Hopkins University Bloomberg School of Public Health, Baltimore, MD USA; 6 Department of Biostatistics, UCLA Fielding School of Public Health, Los Angeles, CA USA; 7 Department of Public Health Sciences, UC Davis School of Medicine, Davis, CA USA; 8 Behavioral, Epidemiologic, and Clinical Sciences, Durham, NC FHI 360 USA; 9 Department of Infectious Disease Epidemiology, London School of Hygiene and Tropical Medicine, London, UK; 10 Zambart, University of Zambia School of Public Health, Lusaka, Zambia; 11 Clinical Research Department, London School of Hygiene and Tropical Medicine, London, UK; 12 Desmond Tutu TB Center, Department of Paediatrics and Child Health, Stellenbosch University, Stellenbosch, Western Cape South Africa; 13 Division of Biostatistics and Bioinformatics, Department of Oncology, Sidney Kimmel Comprehensive Cancer Center, Johns Hopkins University School of Medicine, Baltimore, MD USA

**Keywords:** HIV incidence estimation, Recent infections, Universal antiretroviral treatment, Early antiretroviral treatment, Viral suppression

## Abstract

**Background:**

Multi-assay algorithms (MAAs) are used to estimate population-level HIV incidence and identify individuals with recent infection. Many MAAs use low viral load (VL) as a biomarker for long-term infection. This could impact incidence estimates in settings with high rates of early HIV treatment initiation. We evaluated the performance of two MAAs that do not include VL.

**Methods:**

Samples were collected from 219 seroconverters (infected < 1 year) and 4376 non-seroconverters (infected > 1 year) in the HPTN 071 (PopART) trial; 28.8% of seroconverter samples and 73.2% of non-seroconverter samples had VLs ≤ 400 copies/mL. Samples were tested with the Limiting Antigen Avidity assay (LAg) and JHU BioRad-Avidity assays. Antibody reactivity to two HIV peptides was measured using the MSD U-PLEX assay. Two MAAs were evaluated that do not include VL: a MAA that includes the LAg-Avidity assay and BioRad-Avidity assay (LAg + BR) and a MAA that includes the LAg-Avidity assay and two peptide biomarkers (LAg + PepPair). Performance of these MAAs was compared to a widely used MAA that includes LAg and VL (LAg + VL).

**Results:**

The incidence estimate for LAg + VL (1.29%, 95% CI: 0.97–1.62) was close to the observed longitudinal incidence (1.34% 95% CI: 1.17–1.53). The incidence estimates for the other two MAAs were higher (LAg + BR: 2.56%, 95% CI 2.01–3.11; LAg + PepPair: 2.84%, 95% CI: 1.36–4.32). LAg + BR and LAg + PepPair also misclassified more individuals infected > 2 years as recently infected than LAg + VL (1.2% [42/3483 and 1.5% [51/3483], respectively, vs. 0.2% [6/3483]). LAg + BR classified more seroconverters as recently infected than LAg + VL or LAg + PepPair (80 vs. 58 and 50, respectively) and identified ~ 25% of virally suppressed seroconverters as recently infected.

**Conclusions:**

The LAg + VL MAA produced a cross-sectional incidence estimate that was closer to the longitudinal estimate than two MAAs that did not include VL. The LAg + BR MAA classified the greatest number of individual seroconverters as recently infected but had a higher false recent rate.

**Supplementary Information:**

The online version contains supplementary material available at 10.1186/s12879-022-07850-0.

## Background

Multi-assay algorithms (MAAs) have been developed for estimating population-level HIV incidence using samples collected in cross-sectional surveys [1–3]. These algorithms have also been used to identify individuals with recent HIV infection [4–7]. The most widely used MAA includes the Limiting Antigen Avidity assay (LAg-Avidity assay) and HIV viral load (LAg + VL MAA) [8–10]. In this MAA, low HIV viral load is used as a surrogate for antiretroviral treatment (ART). When this MAA was developed, it was based on the assumption that most persons with low viral loads were on ART and were not recently infected, since ART was only initiated later in infection after CD4 cell count decline [8, 10]. Guidelines for ART have since evolved, and ART is now recommended for all persons living with HIV (PLHIV), regardless of CD4 cell count [11]. The World Health Organization, UNAIDS and other groups have set ambitious targets to increase the number of PLHIV who are aware of their HIV status, are on ART, and are virally suppressed [12].

As early ART initiation becomes more widespread, more persons with recent infection will be virally suppressed. This could lead to underestimation of HIV incidence using MAAs that classify these persons as “non-recent”. This type of misclassification is likely to increase over time with scale-up of universal ART. Furthermore, this type of false non-recent misclassification is likely to vary in different regions and populations based on the proportion of persons with recent HIV infection who are aware of their HIV status and are able to access ART and achieve viral suppression early in infection. For these reasons, accurate HIV incidence estimation may require use of MAAs that do not include viral load. Even when viral load is not included as a biomarker, development of MAAs for use in settings with universal ART is challenging, since viral suppression also impacts the performance of the LAg-Avidity assay and other serologic incidence assays [7, 13–16]. This is because the level and avidity of anti-HIV antibodies often decline when persons are virally suppressed on ART [17].

We recently evaluated the impact of universal ART on the performance of the LAg + VL MAA [7, 18] using samples and data from the HIV Prevention Trials Network (HPTN) 071 (PopART) trial [19]. HPTN 071 (PopART) was a community-randomized trial conducted in Zambia and South Africa that demonstrated that universal delivery of a combination prevention intervention that included universal testing and treatment was associated with decreased HIV incidence [19]. Two years after trial onset, ~ 75% of participants who were infected more than one year and ~ 30% of those who had acquired HIV infection during the study were virally suppressed [19]. The LAg + VL MAA provided an accurate HIV incidence estimate for this cohort [18], but performed poorly for individual-level recency assessments [7].

In this report, we evaluated the performance of two MAAs that do not include HIV viral load. One of these MAAs includes two serologic assays (the LAg-Avidity assay and the BioRad-Avidity assay; LAg + BR MAA). This MAA has provided accurate incidence estimates in some cohorts [20–22], but has not been evaluated in a setting that includes early/universal ART. The second MAA evaluated in this report is a new MAA that includes the LAg-Avidity assay and a novel two-peptide biomarker (LAg + PepPair MAA). We compared the performance of these two MAAs to the performance of the LAg + VL MAA for estimating HIV incidence and identifying recent infections, using samples and data from the HPTN 071 (PopART) trial.

## Methods

### Samples used for the analysis

**Fig. 1:**
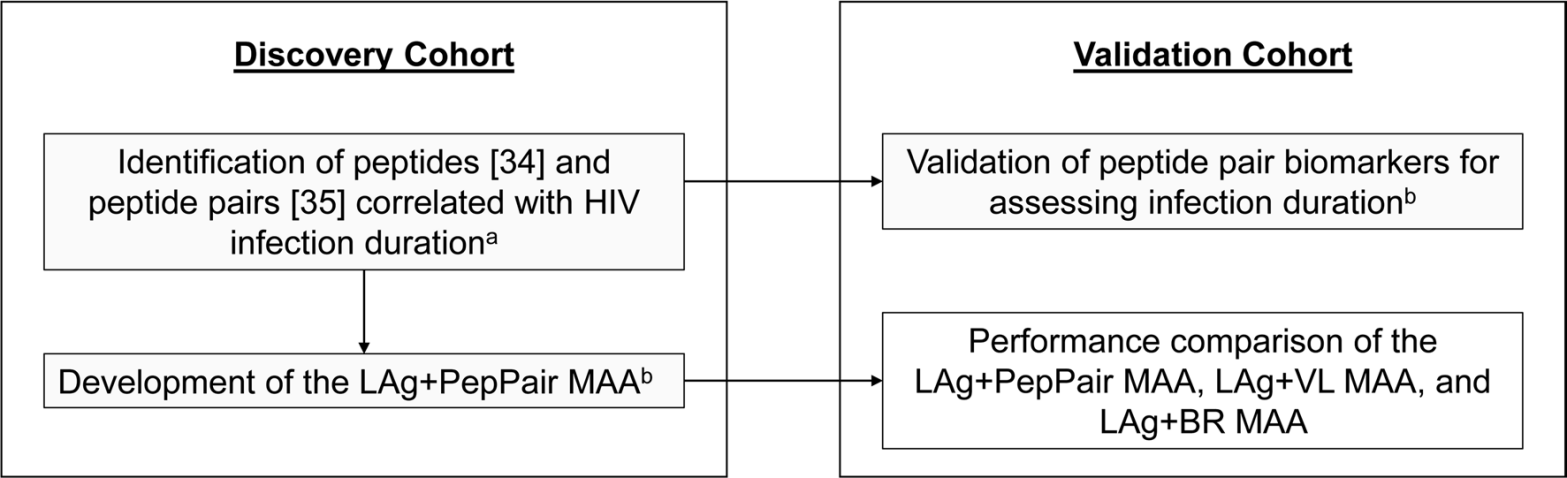
Study design. Samples for this study were obtained from two study cohorts (Table 1). The figure shows the assessments performed with samples from each cohort. Three multi-assay algorithms (MAAs) were assessed (Table 2). ^a^Included in prior publications [25, 26]. ^b^See Additional file 1

This work was performed using samples and data from two cohorts (Table 1, Fig. 1). The Discovery Cohort was used to develop the LAg + PepPair MAA (Additional file 1). This cohort included 324 longitudinal plasma samples from 57 women in Zimbabwe and Uganda who participated in the Hormonal Contraception and HIV Genital Shedding and Disease Progression Study (GS Study, 2001–2009) [23]; this study included women who acquired HIV infection during the Hormonal Contraception and Risk of HIV Acquisition (HC-HIV) Study (NCT00006324; 1999–2002) [24]. ART was initiated according to local treatment guidelines at the time of the study (CD4 cell count < 250 cells/mL) [23, 24]. The date of HIV seroconversion in this sample set was defined as the midpoint between study visits with the last negative and first positive HIV antibody test, or fifteen days after documentation of acute infection (RNA positive, antibody negative). Data from 14 samples were excluded due to unusually low IgG concentration or unusually high background values on the MSD assay. The final Discovery Cohort included 310 samples (201 samples from participants infected < 1 year; 109 samples from participants infected > 1 year).

**Table 1:** Samples used for analysis

Study Cohort	Sample Source	Participant group	Duration of HIV infection	# of samples
Discovery Cohort	Hormonal Contraception and HIV Genital Shedding (GS) Study	Women in Zimbabwe and Uganda (2001–2009), ART provided according to local guidelines CD4 < 250 cells/mL), prevalent subtypes A, C, and D	< 3 months	47
			3–6 months	63
			6–12 months	91
			12–24 months	12
			2–4 years	29
			4–6 years	30
			6–8.7 years	38
Validation Cohort	HPTN 071 (PopART) Study	General population/household survey (2013–2018), men and women, three arm trial including early/universal ART; prevalent subtype C	< 1 year^a^	219
			1–2 years^b^	208
			> 1 year^c^	685
			> 2 years^d^	3483

*ART* antiretroviral therapy

^**a**^Seroconverters (infected < 1 year): HIV negative at PC12; HIV positive at PC24

^**b**^Non-seroconverters (infected 1–2 years): HIV negative at PC0; HIV positive at PC12 and PC24

^**c**^Non-seroconverters (infected > 1 year): HIV status unknown at PC0; HIV positive at PC12 and PC24

^d^Non-seroconverters (infected > 2 years): HIV POS at PC0 and PC24

**Table 2:** Multi-assay algorithms

MAA	Criteria used to classify samples as “recent”	Mean window period
LAg + VL	LAg-Avidity < 1.5 OD-n and VL > 1000 copies/mL	130 (118, 142) [8]
LAg + BR	LAg-Avidity < 2.8 OD-n and BioRad AI < 40%	119 (94, 144) [20]
LAg + PepPair^a^	LAg-Avidity < 2.5 OD-n plus log_10_(peptide pair 4) < − 0.02	102 (69, 159)

The table shows the three multi-assay algorithms (MAAs) evaluated in this report. The mean window period is shown in days; 95% confidence intervals are shown in parentheses

^a^The LAg + PepPair MAA includes the log_10_ ratio of antibody reactivity to two HIV peptides as a biomarker of HIV infection duration. Pep_93621 is a 15-amino acid peptide located in gp160; antibody reactivity to this peptide increases over the course of HIV infection. Pep_92687 is a 22-amino acid peptide located in the gag-polyprotein; antibody reactivity to this peptide decreases over the course of infection. Antibody reactivity to these peptides was measured using the MSD U-PLEX assay. The value included in the MAA (log_10_[peptide pair 4]) was calculated as: log_10_([MSD U-PLEX assay result for upgoing gp160 peptide 93621]/[MSD U-PLEX result for downgoing gag peptide 92687]). Additional file 1 provides additional information on the development and performance of the LAg + PepPair MAA

The Validation Cohort was used to evaluate the performance of peptide biomarkers for use in MAAs (Additional file 1) and to compare the performance of the LAg + VL MAA, the LAg + BR MAA (previously referred to as the Alternate MAA [20]), and the LAg + PepPair MAA. This cohort included plasma samples from participants in the Population Cohort of HPTN 071 (NCT01900977; 2013–2018) [19]. Participants had up to four annual study visits (PC0, PC12, PC24, and PC36). The samples used in this report were collected 2 years after the start of the trial (at PC24; sample collection 2016–2017) from participants who were living with HIV at the PC24 visit and had known HIV status one year earlier (at PC12) [27]. Participants who had acute HIV infection at either visit were excluded from analysis. HIV status at the baseline visit (PC0) was available for a subset of participants; these data were used to identify participants who had been living with HIV for > 2 years at the time of sample collection. Samples were collected from 4452 participants who were HIV positive at both PC12 and PC24 visits (non-seroconverters, infected > 1 year) and 221 participants who were HIV-negative at PC12 and HIV-positive at PC24 (seroconverters, infected < 1 year); 15,845 participants were HIV-negative at both visits.

Fifty-three participants in the non-seroconverter group were excluded from analysis because they were missing data for the LAg-Avidity assay. Twenty-three participants in the non-seroconverter group and two participants in the seroconverter group were excluded due to outlier results for the MSD assay, based on criteria described in Laboratory Methods. The final Validation Cohort included 4376 non-seroconverters and 219 seroconverters; 3483 (79.6%) of the non-seroconverters were known to be infected > 2 years. Sixty-three (28.8%) of the seroconverters and 3205 (73.2%) of the non-seroconverters were virally suppressed at the PC24 visit (viral load ≤ 400 copies/mL).

### Laboratory methods

Testing was performed at the HPTN Laboratory Center (Johns Hopkins University, Baltimore, MD). Testing to determine HIV status and viral load testing were performed previously; viral load testing was performed using the RealTime HIV-1 Viral Load Assay (Abbott Molecular, Des Plaines, IL) with a validated dilution procedure (limit of quantification: 400 copies/mL) [19]. LAg-Avidity values were determined using the HIV-1 Limiting Antigen (LAg)-Avidity EIA (Sedia Biosciences Corporation, Beaverton, OR; LAg-Avidity assay). LAg-Avidity assay results were reported in normalized optical density units (OD-n). The JHU-modified BioRad-Avidity assay was performed as previously described [14]; this assay is based on the Genetic Systems 1/2 + O ELISA (Bio-Rad Laboratories, Redmond, WA). BioRad-Avidity assay results were reported in avidity index values (AI).

Antibody reactivity to HIV peptides was measured using the MSD U-PLEX assay (Meso Scale Discovery; Rockville, MD) [28]. Peptides were synthesized by LifeTein (Somerset, NJ) and Chi Scientific (Maynard, MA). Antibody reactivity was quantified using U-PLEX-10 plates that were coated with HIV peptides (Additional file 1); the ratio of reactivity to two of these peptides (pep_93621 and pep_92687) is used as a biomarker in the LAg + PepPair MAA. Plates were read using the MESO QuickPlex SQ 120 (Meso Scale Discovery; Rockville, MD). Stratified random sampling was used to ensure a balance of samples on each plate with high vs. low viral loads (cutoff log_10_viral load = 2.5) and long vs. short infection duration based on longitudinal follow-up (cutoff: 1 year). Two samples with high vs. low LAg-Avidity values (high: 2.98 OD-n; low: 0.17 OD-n) were included in triplicate on every plate for quality control. Seven or eight wells per plate included reagents only (no plasma samples). Peptides of interest were assigned to individual spots in each well. Additional spots in each well were used to measure signal intensities for the linker only (no peptide attached) and IgG. Four spots in each well were empty and blocked. MSD signal intensities were analyzed on a log_10_ transformed scale. For each plate, the 25th and 75th percentile of the log_10_ transformed signal intensities were calculated for the empty spots, the linker control, and IgG spots; outlier samples with signal intensity values five inter-quartile ranges (IQR) above the 75th percentile or five IQR below the 25th percentile were removed. Antibody reactivity data from the MSD assay were reported as log_10_ ratios of signal intensity of antibody binding for each pair of peptides. Additive plate effects were eliminated using this approach, since the predictors were ratios of measurements from the same well.

### Statistical methods

Data from the Validation Cohort were used to evaluate the performance of three MAAs for cross-sectional HIV incidence estimation and identification of persons with recent HIV infection (the LAg + VL MAA, LAg + BR MAA, and the LAg + PepPair MAA; Table 2). HIV incidence was measured as number of infections per 100 person-years. These estimates were compared to the longitudinal HIV incidence estimate from the HPTN 071 trial between the PC12 and PC24 surveys, which was calculated by dividing observed seroconversions at PC24 by years of follow-up for participants who were HIV-negative at PC12. Incidence estimates and confidence intervals (CIs) were calculated using two methods: first, the method implemented in CEPHIA’s ABIE v3 Incidence Calculator [29], which can be found in the inctools CRAN repository [30], and a second method we have previously described [31–33]. As both methods produced similar results, we present only the results from the first method. The sensitivity and specificity of MAAs for individual-level recency assessments were calculated using known seroconversion status between the PC12 and PC24 visits as the standard for recent vs. non-recent infection. The false recent rate (FRR) was calculated as the percentage of persons classified as recently infected who had been infected > 2 years at the time of sample collection [34]. Univariate analyses were performed using the chi-square test without correction.

### Informed consent

Written informed consent was obtained from participants prior to enrollment in the GS Study and HPTN 071 (PopART) Population Cohort. The GS Study was approved by ethical review boards at FHI 360, Case Western University, University of California, San Francisco and in Uganda and Zimbabwe. The HPTN 071 (PopART) trial was approved by the institutional review board and ethics committees at the London School of Hygiene and Tropical Medicine, the University of Zambia, and Stellenbosch University. The research reported in this manuscript was performed using stored samples and was approved by the Institutional Review Board of the Johns Hopkins University of Medicine. Research was conducted in accordance with the Declaration of Helsinki.

## Results

### Study cohorts

Samples and data from the Discovery Cohort (Table 1) were used to identify peptide biomarkers that provided information on the duration of HIV infection and to develop the LAg + PepPair MAA (Additional file 1). Samples and data from the Validation Cohort (Table 1) were used to validate the performance of ratios of antibody reactivity to paired HIV peptides as biomarkers for assessing HIV infection duration. These samples and data were also used to compare the performance of the LAg + VL MAA to two MAAs that do not include viral load as a biomarker: the LAg + BR MAA and the LAg + PepPair MAA (Table 2).

### LAg + PepPair MAA development

We previously used a multiplexed antibody profiling system (VirScan) to quantify antibody reactivity to > 3300 peptides spanning the HIV genome [25, 35]. We demonstrated that antibody reactivity to specific HIV peptides [25] and ratios of antibody reactivity to paired HIV peptides [26] were correlated with the duration of HIV infection. Peptides identified in those studies were used to develop the LAg + PepPair MAA. A detailed description of the process used for peptide discovery and development of the LAg + PepPair MAA is provided in Additional file 1. Briefly, 11 candidate HIV peptides were identified using VirScan that were differentially targeted by antibodies based on the duration of HIV infection [25, 26]. Four of those peptides were selected for further analysis. Antibody reactivity to synthesized peptides containing corresponding epitopes was quantified using the MSD platform. These data were used to evaluate the performance of 234,000 MAAs that included different combinations of biomarkers (LAg-Avidity, BioRad-Avidity, and/or ratios of antibody reactivity to peptide pairs measured by the MSD assay) with different assay cutoffs. Candidate MAAs were selected that had the highest estimated mean window period with an upper 95% CI for the shadow of < 365 days. The final MAA selected was LAg-Avidity < 2.5 OD-n plus log_10_(peptide pair 4) < − 0.02 (referred to as the LAg + PepPair MAA, Table 2).

### Cross-sectional HIV incidence estimation

**Fig. 2:**
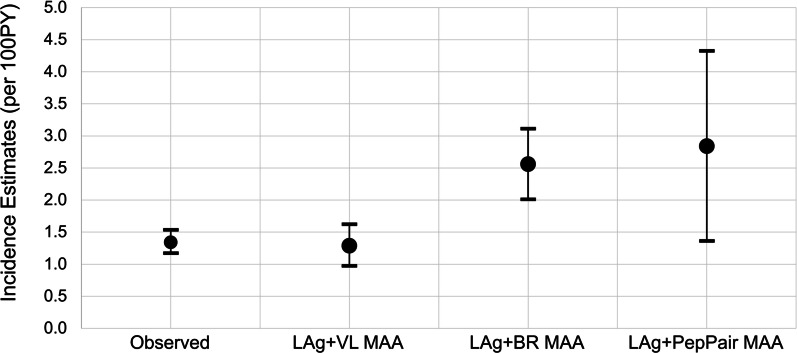
Observed longitudinal Incidence and cross-sectional Incidence estimates, HPTN 071 (PopART). The figure shows the observed longitudinal HIV incidence in the Validation Cohort, compared with incidence estimated using the LAg + VL MAA, the LAg + BR MAA, and the LAg + PepPairA MAA. Error bars represent 95% confidence intervals. *MAA* multi-assay algorithm, *PY* person years

The LAg + VL MAA, LAg + BR MAA and LAg + PepPair MAA were used to estimate HIV incidence using samples collected in HPTN 071 two years after the start of the study intervention (at the PC24 visit). These results were compared to the longitudinal estimate of HIV incidence based on HIV seroconversion in the second year of the trial (between the PC12 and PC24 visits) (Fig. 2). The longitudinal estimate of HIV incidence was 1.34 events per 100 person-years (95% CI: 1.17–1.53) [18]. The incidence estimate for the LAg + VL MAA obtained using the CEPHIA method was very close to the longitudinal incidence estimate (1.29, 95% CI: 0.97–1.62). The incidence estimates using the CEPHIA method for the LAg + BR MAA and LAg + PepPair MAA were much higher (2.56, 95% CI 2.01–3.11 and 2.84, 95% CI: 1.36–4.32, respectively).

### Identification of individuals with recent infection

We next compared the performance of the three MAAs for identifying persons with recent infection (seroconverters, infected < 1 year, Table 3). The three MAAs classified different numbers of the 219 seroconverters as recently infected (LAg + VL MAA: 58 [26.5%]; LAg + BR MAA: 80 [36.5%]; LAg + PepPair MAA: 55 [25.1%]). Note that the LAg + BR MAA identified the highest number of participants as recently infected, even though the window period of this MAA was lower than that of the LAg + VL MAA (LAg + VL MAA: 130 days; LAg + BR MAA: 119 days). The three MAAs also misclassified different numbers of the 4376 non-seroconverters as recent (LAg + VL MAA: 11 [0.3%]; LAg + BR MAA: 65 [1.5%]; LAg + PepPair MAA: 71 [1.6%]). The FRR (percentage of individuals infected > 2 years classified as recent) was 0.2%, 1.2%, and 1.5% for the LAg + VL MAA, the LAg + BR MAA, and the LAg + PepPair MAA, respectively.

**Table 3:** Performance of multi-assay algorithms for identifying individuals with recent HIV infection

	Classification	LAg + VL MAA^a^	LAg + BR MAA	LAg + PepPair MAA
Seroconverters infected < 1 year (N = 219)	MAA recent	58	80	55
	MAA non-recent	160	139	164
Non-seroconverters infected > 1 year (N = 4376)	MAA recent	11	65	71
	MAA non-recent	4365	4311	4305
Non-seroconverters infected > 2 years (subset, N = 3483)	MAA recent	6	42	51
	MAA non-recent	3477	3441	3432
Sensitivity^b^		26.5%	36.5%	25.1%
Specificity^b^		99.7%	98.5%	98.4%
Positive predictive value^b^		84.1%	55.2%	43.7%
Negative predictive value^b^		96.4%	96.9%	96.3%
False recent rate^c^		0.2%	1.2%	1.5%

The table shows the performance characteristics for three multi-assay algorithms (MAAs) for identifying individuals with recent HIV infection (infected < 1 year). The cohort included 219 persons infected < 1 year (seroconverters) and 4376 persons infected for > 1 year (non-seroconverters). Sixty-three (28.8%) of the seroconverters and 3205 (73.2%) of the non-seroconverters were virally suppressed (viral load ≤ 400 copies/mL)

^a^One participant did not have a viral load result and could not be classified as recent vs. nonrecent by the LAg+VL MAA

^b^Sensitivity, specificity, positive predictive value, and negative predictive value were calculated classifying recent infection as <1 year since the last negative HIV test

^c^The false recent rate indicates the proportion of participants infected > 2 years who were classified as recently infected

Only 24 (11.0%) of the seroconverters were classified as recently infected by all three MAAs. This is consistent with a prior report that showed that different MAAs identify different subsets of seroconverters as recently infected [7]; the different classification of individual seroconverters as recently infected was not solely due to exclusions for viral load. In this study, 80 of the seroconverters were classified as recent by the LAg + BR MAA. Only 47 (59%) of those participants were also classified as recent by the LAg + VL MAA; 19 of the remaining 33 participants were excluded based on viral load. An overlapping subset of 55 seroconverters ws classified as recent by the LAg + PepPair MAA. Only 29 (53%) of those participants were also classified as recent using the LAg + VL MAA; 20 of the remaining 26 participants were excluded based on viral load.

### Impact of viral suppression on MAA performance

By design, the LAg + VL MAA classified all participants with viral loads < 1000 copies/mL as having non-recent infection (Table 4). Viral suppression (viral load ≤ 400 copies/mL) significantly impacted the performance of the LAg + BR MAA but not the LAg + PepPair MAA for classifying seroconverters as recently infected (virally suppressed vs. viremic: 25.4% [16/63] vs. 40.6% [63/155], p = 0.034 for the LAg + BR MAA; 30.2% [19/63] vs. 22.6% [35/155], p = 0.24 for the LAg + PepPair MAA). The majority of the non-seroconverters who were misclassified as recently infected with the LAg + BR MAA and LAg + PepPair MAA were virally suppressed (LAg + BR MAA: 56/65 [86.2%]; LAg + PepPair MAA: 69/71 [97.2%]). However, these persons represented a small proportion of all suppressed non-seroconverters (LAg + BR MAA: 56/3205 [1.7%]; LAg + PepPair MAA: 69/3205 [2.2%]). The performance of the LAg + BR MAA and LAg + PepPair MAA for classifying seroconverters as recently infected was also not impacted by age or gender.

**Table 4:** Performance of multi-assay algorithms for classifying seroconverters as recently infected stratified by viral suppression status, age, and gender

		LAg + VL MAA^a^			LAg + BR MAA			LAg + PepPair MAA		
	Total N = 219	Recent N = 58	Nonrecent N = 160	P-value	Recent N = 80	Nonrecent N = 139	P-value	Recent N = 55	Nonrecent N = 161	P-value
VL status^b^				** < 0.0001**			**0.034**			0.24
Suppressed	63	0 (0.0%)	63 (100.0%)		16 (25.4%)	47 (74.6%)		19 (30.2%)	44 (69.8%)	
Viremic	155	58 (37.4%)	97 (62.6%)		63 (40.6%)	92 (59.4%)		35 (22.6%)	120 (77.4%)	
Age (years)				0.57			0.54			0.78
18–24	88	21 (23.9%)	67 (76.1%)		30 (34.1%)	58 (65.9%)		23 (26.1%)	65 (73.9%)	
25 +	131^a^	37 (28.5%)	93 (71.5%)		50 (38.2%)	81 (61.8%)		32 (24.4%)	99 (75.6%)	
Gender				0.65			0.67			0.83
Female	189^a^	49 (26.1%)	139 (73.9%)		68 (36.0%)	121 (64.0%)		47 (24.9%)	142 (75.1%)	
Male	30	9 (30.0%)	21 (70.0%)		12 (40.0%)	18 (60.0%)		8 (26.7%)	22 (73.3%)	

The table shows the performance of the LAg + VL MAA, LAg + BR MAA, and LAg + PepPair MAA for classifying the 219 seroconverters from the Validation Cohort as recently infected, stratified by viral suppression status, age, and gender. P-values shown in bold font are statistically significant

^a^One participant (female, 25+ years) did not have viral load data and could not be classified as recent vs. nonrecent by the LAg+VL MAA

^b^Viral suppression was defined as having a viral load ≤ 400 copies/mL; one participant did not have viral load determined

## Discussion

This study compared the performance of MAAs for cross-sectional HIV incidence estimation and individual recency assessments using samples and data from HPTN 071 (PopART), a cohort with substantial rates of viral suppression and early ART. This comparison included a widely used MAA that includes viral load (LAg + VL MAA), and two MAAs that do not include viral load (LAg + BR MAA, LAg + PepPair MAA). Despite high levels of ART-induced viral suppression, the LAg + VL MAA provided a point estimate of HIV incidence for this cohort that was nearly identical to the incidence estimate obtained by longitudinal follow-up of the HPTN 071 (PopART) cohort (LAg + VL: 1.29%; longitudinal: 1.34%) [18]. The discrepancy between the performance of this MAA for classifying individuals as recently infected and estimating incidence at a population level may reflect the fact that additional performance characteristics impact population-level incidence estimates (e.g., false recent rate among those with non-recent HIV infection). The two MAAs that did not include viral load provided overestimated incidence predictions (LAg + BR MAA: 2.56%; LAg + PepPair MAA: 2.84%). Of note, while we did obtain a high point estimate of incidence using the LAg + PepPair MAA, the 95% confidence interval for this MAA (1.36–4.32) overlapped with the 95% confidence intervals for the longitudinal incidence estimate (1.17–1.53) and the incidence estimate obtained using the LAg + VL MAA (0.97–1.62).

All three MAAs performed poorly for individual-level recency assessments. The sensitivity for the three MAAs ranged from 25.1% to 36.5%; the low sensitivities, in part, reflect the mean window periods for the MAAs, which range from 102 to 130 days. Of note, the LAg + BR MAA had a shorter mean window period than the LAg + VL MAA (119 vs. 130 days), but identified a higher number of seroconverters as recently infected (80 vs. 58). However, the false recent rate was higher for the LAg + BR MAA than the LAg + VL MAA (1.2 vs. 0.2%). This illustrates the trade-off between identifying more recently infected individuals (sensitivity) and misidentifying those with long-term infection as recently infected (specificity).

By design, the LAg + VL MAA classified all virally suppressed seroconverters as not recently infected. While viral load significantly impacted the identification of seroconverters as recently infected using the LAg + BR MAA, this MAA still classified ~ 25% of virally suppressed seroconverters as having recent infection. The LAg + BR MAA also correctly classified a higher number of all seroconverters as recently infected compared to the LAg + VL MAA (80 vs. 58). As ART coverage continues to improve, there will be an increasing role for MAAs that do not include viral load as a biomarker and are not impacted by viral suppression. Further research is needed to develop MAAs that provide accurate incidence estimates in those settings. While the LAg + BR MAA did not perform well for cross-sectional incidence estimation, it performed better than the other MAAs for individual-level recency assessments in this cohort. Even so, the low positive predictive value of this MAA (55%) indicates that further research is needed to identify accurate testing approaches for individual-level assessments in settings with high levels of early ART initiation.

Despite our efforts to identify new peptide biomarkers for recency assessments, a MAA that included top-performing peptide biomarkers (LAg + PepPair MAA) did not accurately estimate incidence in the HPTN 071 (PopART) cohort. The sample set used to develop the LAg + PepPair was much smaller than the sample set used to develop the LAg + BR MAA (310 vs. 1782 samples) [20], which likely accounted for the broad confidence intervals for the LAg + PepPair MAA. MAAs that provide precise incidence estimates with narrow confidence intervals are needed for estimating HIV incidence, which usually ranges from 1–3%; further analyses using a larger training sample set are needed to fully assess the performance of the PepPair MAA.

While viral suppression did not impact the identification of seroconverters as recently infected using the LAg + PepPair MAA, this MAA still identified fewer seroconverters as recently infected than the LAg + VL MAA or the LAg + BR MAA (50 vs. 58 and 80) and had the highest FRR (1.5%) among the MAAs evaluated. The difference in the FRRs obtained for this MAA in the Discovery and Validation cohorts might reflect demographic or other differences between the populations.

One limitation of this study is that the Discovery Cohort used for development of the LAg + PepPair MAA did not include any participants who were receiving early ART; in contrast 28.8% of the participants in the Validation Cohort were virally suppressed on ART within the first year of infection. Additionally, the Discovery Cohort included participants with HIV subtypes A, C, and D, while the Validation Cohort included participants with only HIV subtype C. Genetic diversity between subtypes or local HIV strains could potentially influence the performance of peptide-based MAAs. Further validation of these approaches is needed in cohorts with different HIV subtypes to determine the generalizability of these methods. Also, the VirScan assay that was used for peptide discovery only measures antibody reactivity to linear, non-glycosylated peptides; conformational and/or glycosylated HIV epitopes may serve as better biomarkers for estimating the duration of HIV infection. We are also exploring whether antibody reactivity to peptides in the HIV antisense protein [36] could be a useful biomarker for HIV incidence estimation; those peptides were not included in the VirScan phage library used in this report. Finally, study visits for the HPTN 071 (PopART) trial were conducted annually. For calculations of sensitivity and specificity, true positive cases included seroconverters with known infection duration < 1 year who were classified as recently infected. As all three MAAs have mean window periods < 6 months, only a fraction of such individuals would be expected to be classified as recent. Window periods vary between different populations and cohorts [8, 33, 34, 37–39], and other factors, such as false recent rate and recency distribution profiles, also impact MAA performance. These factors should be considered when evaluating MAA performance.

## Conclusion

Two MAAs that did not include viral load as a biomarker did not provide accurate incidence estimates in this study. The LAg + BR MAA was more robust than the widely used LAg + VL MAA for identifying individuals with recent HIV infection and identified many of these persons as recently infected despite viral suppression. Accurate MAAs are needed to track the leading edge of the HIV epidemic and target resources for HIV prevention. The accuracy of MAAs may also impact interpretation of clinical trials and evaluation of HIV prevention programs. Further research is needed to identify robust MAAs that do not include viral load for analysis of populations where most recently infected persons are virally suppressed on ART early in infection.

## Supplementary Information

**Additional file 1.** Development of the LAg + PepPair MAA.

## Data Availability

The datasets used and/or analyzed during the current study are available from the corresponding author on reasonable request.

## Abbreviations

AI: Avidity index
ART: Antiretroviral treatment
BR: BioRad-Avidity assay
CI: Confidence interval
FRR: False recent rate
GS Study: Hormonal Contraception and HIV Genital Shedding and Disease Progression Study
HC-HIV Study: Hormonal Contraception and Risk of HIV Acquisition Study
HPTN: HIV Prevention Trials Network
IQR: Inter-quartile range
LAg: Limiting Antigen Avidity assay
MAA: Multi-assay algorithm
MSD: Meso Scale Discovery
OD-n: Optical density units
PLHIV: Persons living with HIV
PY: Person years
VL: Viral load
