# Supplementary material for: Evaluation of multi-assay algorithms for cross-sectional HIV incidence estimation in settings with universal antiretroviral treatment
Source: BMC Infect Dis. 2022 Nov 11;22:838. doi: 10.1186/s12879-022-07850-0 (PMC9652879; doi:10.1186/s12879-022-07850-0)
Supplement: Supplementary file 1 — Additional file 1. Development of the LAg + PepPair MAA. [file 12879_2022_7850_MOESM1_ESM.docx]

**Additional file 1: S1. Development of the LAg+PepPair MAA**

Candidate peptides for use in multi-assay algorithms (MAAs) for cross-sectional HIV incidence estimation were identified using a multiplexed antibody profiling system (VirScan) that quantifies antibody binding to overlapping 56-amino acid peptides expressed on bacteriophage. The VirScan phage library includes >3,300 HIV peptides spanning the viral genome. Using samples from the Discovery Cohort, we identified 11 HIV peptides that were differentially targeted by antibodies based on the duration of HIV infection (Table S1) [25, 26]. The software epitopefindr (<https://github.com/brandonsie/epitopefindr>) was used to identify the likely epitope for each peptide; 10 unique epitopes were identified for the 11 peptides (Table S1). Two peptides were evaluated that had the same epitope but differed in length (pep_92687, short and long). One peptide with a duplicate epitope (pep_32119) was excluded from further analysis.

**Table S1. Candidate peptides identified using the VirScan assay and epitopefindr.**

| **Peptide Name** | **Location (epitope length)** | **Expression^a^** | **Peptide Sequence^b^** | **UniProt ID number** | **HBX2 Coordinates^c^** |
| --- | --- | --- | --- | --- | --- |
| pep_93864 | Gag_polyprotein (55) | ↑ | GT**YRIKHLVWASRELDRFALNPGLLESAKGCQQILVQLQPALQTGTQEIKSLYNTVA**TT | Q9WC53 | 874-1041 |
| pep_20904 | Gag-Pol (46) | ↓ | QE**VKGWMTDTLLVQNANPDCKTILRALGPGATLEEMMTACQGVGGPSH**KA | O41798 | 1720-1869 |
| pep_32119 | Gag-Pol (46) | ↓ | QE**VKGWMTDTLLVQNANPDCKTILRALGPGATLEEMMTACQGVGGPSH**KA | P0C1K7 | 1720-1869 |
| pep_77241 | Gag_polyprotein (46) | ↓ | QD**VKNWMTDTLLVQNANPDCKTILRALGPGASLEEMMTACQGVGGPAH**KA | Q75001 | 1720-1869 |
| pep_92687-short | Gag_polyprotein (22) | ↓ | CSE**RQANFLGKFWPLNKERPGNFLQ**NRPEPTAPPAESFGFGEKITPSLRQEMKDQE | Q9QC00 | 2065-2235 |
| pep_92687-long | Gag_polyprotein (28) | ↓ | **CSERQANFLGKFWPLNKERPGNFLQNRP**EPTAPPAESFGFGEKITPSLRQEMKDQE | Q9QC00 | 2065-2235 |
| pep_25409 | Gag-pol (33) | ↑ | RV**YYRDSRNPLWKGPAKLLWKGEGAVVIQDNSDIK**VV | P03366 | 4899-5009 |
| pep_26777 | Gag-Pol (33) | ↑ | RV**YYRDNRDPIWKGPAKLLWKGEGAVVIQDNSDIK**VV | P04588 | 4899-5009 |
| pep_93621 | Gp160 (15) | ↑ | NGSLAEEGIVIRSQNISNNAKTIIVHLNESVQINCTRPNNN**TRKGIHLGPGQTFYA** | Q9QSQ7 | 7008-7175 |
| pep_77323 | Gp160 (29) | ↑ | IR**IGPGQTFYATGDIIGDIRQAHCNISEEKW**NK | Q75008 | 7149-7241 |
| pep_49812 | Gp160 (15) | ↑ | AQQHLLQLTVWGIKQLQARVLAVERYLRDQQ**LLGIWGCSGKLICTTT**VPWNASWSN | P31872 | 7905-8072 |

^a^ Arrows indicate the change in antibody reactivity as a function of duration of HIV infection. An up arrow indicates that antibody reactivity tends to increase over time. A down arrow indicates that antibody reactivity tends to decrease over time.

^b^ The likely epitope sequence for each peptide is shown in bold.

^c^ HXB2 coordinates are shown for reference strain NCBI #NC_001802.

Shorter versions of the peptides that contained the epitopes of interest were synthesized by LifeTein (Somerset, NJ) and Chi Scientific (Maynard, MA). Synthesis was successful for nine of the ten remaining candidate peptides. Antibody reactivity to the synthesized peptides was measured using the MSD assay. These results were compared to results obtained for antibody reactivity to the 56-amino acid “parent” peptides in the VirScan assay (Figure S1).

**Figure S1. Comparison of antibody reactivity measured with the MSD and VirScan assays.**

The figure shows scatter plots comparing MSD values (relative light units, RLU) to VirScan fold change values for nine of the peptides shown in Table A (pep_25409 had low antibody reactivity on the VirScan assay; pep_32119 shared a duplicate epitope with pep_20904; pep_20904 could not be synthesized). This analysis was performed using 88 samples from the Discovery Cohort. For VirScan analysis, plasma samples containing 2 ug of IgG were analyzed. For MSD analysis, plasma samples were diluted 1:33,000.

Based on the analysis shown in Figure S1, four of the nine candidate peptides were excluded due to low signal strength and thus poor data correlation. Two of the remaining five peptides had the same epitope, but one peptide was longer; the longer peptide was excluded. Table S2 shows the original set of 12 candidate peptides identified by VirScan, the reason for exclusion of eight peptides, and the four peptides that were used for further analysis.

**Table S2. Selection of peptides for further analysis.**

| **Peptide** | **Expression**^a^ | **Reason for exclusion** | **Selected for analysis** |
| --- | --- | --- | --- |
| pep_93864 | ↑ | Poor MSD correlation with VirScan^b^ |  |
| pep_20904^c^ | ↓ | Synthesis failed |  |
| pep_32119^c^ | ↓ | Duplicate epitope |  |
| **pep_77241** | ↓ |  | Yes |
| **pep_92687-short^d^** | ↓ |  | Yes |
| pep_92687- long^d^ | ↓ | Duplicate peptide |  |
| pep_25409 | ↑ | Poor MSD correlation with VirScan^b^ |  |
| pep_26777 | ↑ | Poor MSD correlation with VirScan^b^ |  |
| **pep_93621** | ↑ |  | Yes |
| pep_77323 | ↑ | Poor MSD correlation with VirScan^b^ |  |
| **pep_49812** | ↑ |  | Yes |

The 11 candidate peptides are shown. Eight of the 11 peptides were excluded from analysis, for the reasons noted. The four peptides included in the analysis are shown in bold font.

^a^ Arrows indicate the change in antibody reactivity as a function of duration of HIV infection. An up arrow indicates that antibody reactivity tends to increase over time. A down arrow indicates that antibody reactivity tends to decrease over time.

^b^ The MSD signal intensity for these peptides was low and not correlated with antibody reactivity as measured with the VirScan assay (see Figure S1).

^c^ These two peptides had the same predicted epitope; pep_20904 was selected for synthesis.

^d^ These two peptides were derived from the same sequence but with two different predicted epitopes; pep_92687- long is 6 amino acids longer than pep_92687-short; the shorter candidate epitope (22 aa) was selected for analysis.

As a first step, we evaluated MAAs that included different combinations of assays (the LAg-Avidity assay, the BioRad-Avidity assay, and MSD data for one or more peptides), using a range of cutoffs for each assay; none of those MAAs had acceptable performance characteristics. However, in a prior study, we demonstrated that the association between antibody reactivity to HIV peptides and duration of infection may be enhanced when reactivity is assessed for peptide pairs [26]. Based on those findings, we next evaluated the performance of MAAs that included peptide pairs. For this analysis, the four peptides selected above were grouped into four pairs (Table S3); each peptide pair included one up-going peptide (with increased antibody targeting over the course of infection) and one down-going peptide (with decreased antibody targeting over the course of infection). Antibody reactivity data from the MSD assay was reported as log_10_ ratios of signal intensity of antibody binding for each pair of peptides.

**Table S3. Assignment of candidate peptides to peptide pairs.**

| **Pair** | **Up-going peptide** | **Down-going peptide** |
| --- | --- | --- |
| 1 | pep_49812 | pep_77241 |
| 2 | pep_49812 | pep_92687 |
| 3 | pep_93621 | pep_77241 |
| 4 | pep_93621 | pep_92687 |

Data from the Validation Cohort were then used to determine if the ratio of MSD-measured antibody reactivity to each peptide pair was correlated with recent vs. nonrecent HIV infection in an independent data set. These data were used to calculate sensitivity (for duration of infection <1 year) and specificity (against duration of infection >1 year) for the range of all possible cut-off values of the log_10_ ratio of signal intensity of reactivity for each peptide pair. Instead of using a conventional receiver operating characteristic (ROC) curve, sensitivity and specificity were plotted as functions of the cut-off to determine the cutoff for the peptide pair. We then assessed whether the MSD signal intensity ratios for any of the four peptide pairs could serve as informative biomarkers of recent HIV infection (Figure S2).

**Figure S2. Performance of peptide pair log_10_ ratios of antibody reactivity as biomarkers for indicating duration of HIV infection.**

The figure shows non-conventional ROC curves illustrating sensitivity for duration of infection <1 year (blue line) and specificity against duration of infection >1 year (red line) as a function of the biomarker cut-off value (log_10_ ratio of signal intensity for each peptide pair). These curves were used to inform cut-point selection, validating an optimal cut-point of < -0.02 for log_10_(pair 4) for inclusion as an informative biomarker for duration of infection.

We next evaluated MAAs that included ratios of antibody reactivity for peptide pairs as potential biomarkers of HIV infection duration using samples and data from the Discovery Cohort. The analysis shown in Figure S2 illustrated that peptide pair 4 was the most informative biomarker for assessing duration of infection, using a cutoff of log_10_(pair 4) < -0.02. This biomarker was included in all MAAs evaluated below.

As a final step, we evaluated a set of MAAs (N=234,000) that included log_10_(pair 4) < -0.02 and one or more of the following biomarkers: LAg-Avidity assay, BioRad-Avidity assay, and antibody reactivity ratios for the other three peptide pairs. The MAAs evaluated included all possible biomarker combinations, with different cutoffs for each biomarker. The candidate cutoffs for the log_10_ ratio of peptide pair signal intensity were chosen as the 10%-90% deciles of the empirical distributions, rounded to one significant digit. As in previous work [33], twenty-six cut-offs were evaluated for the LAg-Avidity assay (0.5 OD-n to 3.0 OD-n in increments of 0.1 OD-n) and eight cutoffs were evaluated for the BioRad-Avidity assay (AI: 30%, 35%, 40%, 80%, 85%, 90%, 95% and 100%); cutoffs between 40% and 80% were not evaluated due to poor assay reproducibility in this range.

Since all biomarkers under consideration increased with infection duration, candidate MAAs classified samples as positive (indicating recent infection) when all biomarkers were below their respective cutoffs. To avoid overfitting and reduce the number of algorithms processed, 9,839 MAAs were excluded from further analysis because they produced data sets containing fewer than 5 positive or 5 negative classifications. For the remaining MAAs, logistic regression models with cubic polynomials were used to estimate $\phi(t)$, the probability of an MAA-positive test result given duration of infection $T=t$. To fit these models, the dates of infection (defined above) were imputed 1000 times for each participant from uniform distributions over the infection censoring windows, and the coefficients of the resulting $\hat{\phi}(t)$ curves were averaged to produce a final $\hat{\phi}(t)$ estimate.

MAAs that identified more than 1 in 1,000 samples collected >8 years after infection as recent were removed from further consideration. For the remaining MAAs (N=224,161), the $\hat{\phi}(t)$ function was used to calculate the corresponding mean window period (the average duration of time an individual was MAA positive) and shadow (how far back in time the MAA estimates incidence) (Figure C). Confidence intervals (CIs) for the mean window period and shadow were computed using a bootstrap procedure that was stratified by cohort and clustered by individual to account for correlations between samples drawn from the same person over time. Each bootstrap iteration used 100 imputations of the infection date, for the sake of computational speed. Optimal MAAs were selected that had the highest estimated mean window period, among the MAAs that had an upper 95% CI for the shadow of <365 days (Table S4). Because the difference in mean window periods between the highest performing MAAs was small, we selected one that included the smallest number of biomarkers. In this MAA, samples were identified as “recent” if they had the following test results: LAg-Avidity <2.5 OD-n plus log_10_(pair 4) < -0.02. This MAA is referred to in the manuscript as the LAg+PepPair MAA.

**Figure S4: Estimates of mean window period and shadow.**

**
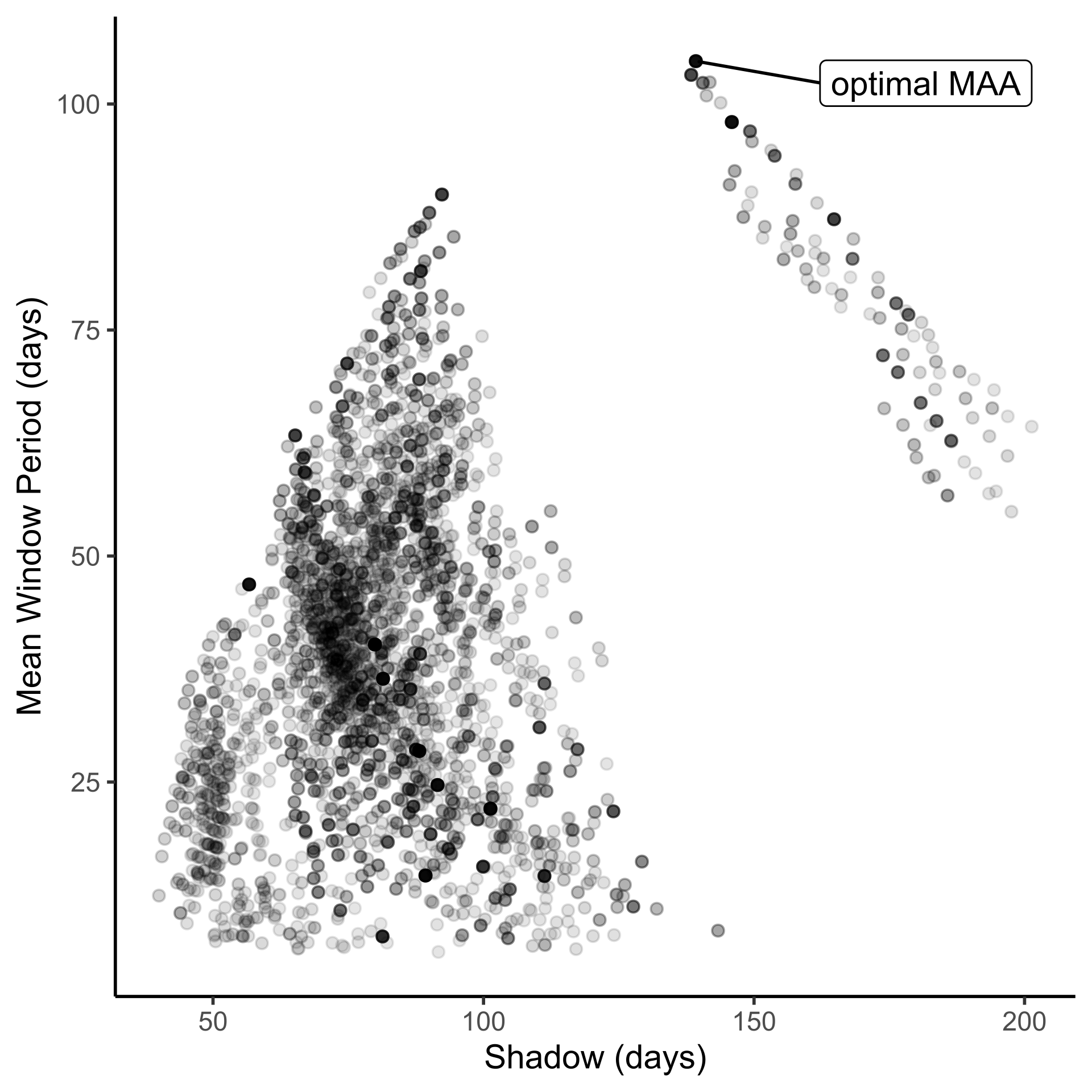
**

The figure shows the mean window period and shadow for each of the 224,161 multi-assay algorithms (MAAs) evaluated. Each dot represents one or more MAAs with the same performance characteristics; darker dots represent larger sets of MAAs with the same characteristics. Nine of the 10 top scoring MAAs had equivalent performance characteristics. Each of these MAAs has a mean window period of 104.7 days and a shadow of 139.2 days. The MSD-MAA that was selected for further analysis (LAg-Avidity <2.5 OD-n, log_10_(pair 4) < -0.02) has a mean window period of 102 days and a shadow of 141 days.

**Table S4: Top performing MAAs that include the LAg-Avidity assay and log_10_(pair4) with a cutoff of -0.02.**

|  | **Biomarkers included** | | | | | | **Performance characteristics** | | | | | |
| --- | --- | --- | --- | --- | --- | --- | --- | --- | --- | --- | --- | --- |
| # of MAAs in group | BioRad | LAg | log_10_  (pair 1) | log_10_  (pair 2) | log_10_  (pair 3) | log_10_  (pair 4) | Mean WP | Mean WP 2.5% | Mean WP 97.5% | Shadow | Shadow 2.5% | Shadow 97.5% |
| 9 | * | ** | 1.0 | 1.5 | 0.9 | -0.02 | 104.7 | 72.37 | 158.0 | 139.2 | 80.86 | 288.9 |
| 9 | * | ** | 2.3 | – | 0.5 | -0.02 | 104.7 | 72.37 | 158.0 | 139.2 | 80.86 | 288.9 |
| 9 | * | ** | – | 2.7 | 0.5 | -0.02 | 104.7 | 72.37 | 158.0 | 139.2 | 80.86 | 288.9 |
| 900 | * | *** | † | †† | ††† | -0.02 | 103.2 | 70.82 | 158.6 | 138.4 | 78.08 | 295.6 |
| 270 | * | ** | † | †† | 0.2 | -0.02 | 102.4 | 69.96 | 155.2 | 141.8 | 77.22 | 304.4 |
| **450** | ***** | **2.5** | **†** | **††** | **†††** | **-0.02** | **102.3** | **69.33** | **158.8** | **140.5** | **76.61** | **299.2** |
| 180 | * | *** | † | †† | 0.2 | -0.02 | 100.9 | 68.70 | 155.8 | 141.2 | 74.77 | 314.1 |

The table shows performance characteristics of the top performing MAAs (7 groups representing 1,827 MAAs). The candidate MAAs included log_10_(pair 4) < -0.02 and one to five additional biomarkers. MAAs included different combinations of biomarkers with different cut-offs (see footnotes). All of the MAAs in each group have the same performance characteristics. The LAg+PepPair MAA is one of the MAAs in the set shown in bold; that MAA was selected because it included the smallest number of biomarkers (the LAg-Avidity assay and one peptide pair). For this group of MAAs, inclusion of additional data for the BioRad-Avidity assay and/or the other three peptide pairs using the selected cutoffs did not impact performance.

“–“ indicates that a biomarker was not included

^*^ A single asterisk indicates that the BioRad-Avidity assay was included with a cutoff of 95 or 100 avidity index (AI), or was not included.

^**^ A double asterisk indicates that the LAg-Avidity assay was included with a cutoff of 2.8, 2.9, or 3.0 normalized optical density units (OD-n).

^***^ A triple asterisk indicates that the LAg-Avidity assay was included with a cutoff of LAg = 2.6 or 2.7.

^†^ This symbol indicates that log_10_(pair 1) was included with a cutoff of 1.0, 1.5, 1.9, or 2.3, or was not included.

^††^ This symbol indicates that log_10_(pair 2) was included with a cutoff of 1.5, 1.7, 2.0, 2.4, or 2.7, or was not included.

^†††^ This symbol indicates that log_10_(pair 3) was included with a cutoff of 0.5, 0.9, 1.4, or 2.0, or was not included.

Abbreviations: LAg: LAg-Avidity assay (normalized optical density units [OD-n]); BioRad: BioRad-Avidity assay (avidity index); WP: window period (in days).
